# Mental Blindness

**Published:** 1890-02-08

**Authors:** 


### MENTAL BLINDNESS.

This term, "Seelenblindheit," has been given in Germany to a peculiar affection related to aphasia, but attended apparently in all cases by hemianopsia, the diagnosis of which demands a careful examination of the acuity of vision, and of the perception of colours as well as of the intensity of light. A typical case is reported by Dr. Lissauer, in the person of a man 80 years of age, who had latterly been subject to "fainting fits." After one of these his family noticed that though his eyesight did not appear to be impaired he could no longer find many articles, even the most familiar, in his room. Dr. Lissauer, after putting him through a careful examination, ascertained that it was only with objects for the recognition of which he was wholly dependent on his eyesight, that he experienced this difficulty, for there was none when he could avail himself of the assistance afforded by touch, or the senses of hearing and smell. He could see well enough, though his acuity was reduced to one-third, and he had complete and absolute right hemianopsia. There was no evidence of aphasia, or other cerebral lesion, and he could distinguish colours with precision. He retained the power of writing, though at first he lost that of reading entirely, but soon improved in this respect. It thus appeared that the optical recollection of recent impressions was more impaired than that of past.

We shall not attempt to follow Dr. Lissauer in his psychological and physiological speculations as to the nature and causes of this defect, in which he seems to have got out of his depth, but compare with his case one reported in the same journal ("Arch. für Psychiatrie"), by Dr. Siemerling. In this a healthy man, aged 54, was suddenly struck down with vertigo but without loss of consciousness, followed by a peculiar disturbance of vision, which at first sight looked like "Seelenblindheit." Like Dr. Lissauer's patient he could see, but could recognize nothing rightly until he had invoked the aid of touch or other senses, and even then he could not always give the object its proper name. He was, therefore, evidently the subject of a form of amnesic aphasia. He was completely hemianopsic on the right side, and partially so on the left, while his acuity of vision was reduced to one-thirtieth. Sensibility to light was fairly perfect, but though both the posterior structures of the eye and its refracting media were intact the perception of colours was entirely lost, and he was perfectly monochromatic. Subsequently he improved greatly in every respect, even to the extent of recovering some appreciation of colours, and of recognizing objects by sight alone. Dr. Siemerling thinks that his case tends to support Wilbrand's assumption of different regions of the cortex being appropriated to the several perceptions of light colour, form or existence in space, &c.

